# Circulating adipokine levels and COVID-19 severity in hospitalized patients

**DOI:** 10.1038/s41366-022-01246-5

**Published:** 2022-12-12

**Authors:** Antine W. Flikweert, Anneke C. Muller Kobold, Simone van der Sar-van der Brugge, Peter Heeringa, Izabela A. Rodenhuis-Zybert, Johan Bijzet, Adriana Tami, Bernardina T. F. van der Gun, Karin I. Wold, Anke Huckriede, Hildegard Franke, Judith M. A. Emmen, Marloes Emous, Marco J. J. H. Grootenboers, Matijs van Meurs, Peter H. J. van der Voort, Jill Moser

**Affiliations:** 1 Department of Critical Care, University of Groningen, University Medical Center Groningen, Groningen, The Netherlands; 2 Department of Pulmonary Medicine, Amphia Hospital, Breda, The Netherlands; 3 Department of Laboratory Medicine, University of Groningen, University Medical Center Groningen, Groningen, The Netherlands; 4 Department of Pathology and Medical Biology, University of Groningen, University Medical Center Groningen, Groningen, The Netherlands; 5 Department of Medical Microbiology & Infection Prevention, University of Groningen, University Medical Center Groningen, Groningen, The Netherlands; 6 Department of Rheumatology & Clinical Immunology, University of Groningen, University Medical Center Groningen, Groningen, The Netherlands; 7 Result Laboratory, Amphia Hospital, Breda, The Netherlands; 8 Center Obesity Northern Netherlands (CON), Department of Surgery, Medical Center Leeuwarden, Leeuwarden, The Netherlands

**Keywords:** Translational research, Obesity

## Abstract

**Background:**

Obesity is a risk factor for adverse outcomes in COVID-19, potentially driven by chronic inflammatory state due to dysregulated secretion of adipokines and cytokines. We investigated the association between plasma adipokines and COVID-19 severity, systemic inflammation, clinical parameters, and outcome of COVID-19 patients.

**Methods:**

In this multi-centre prospective cross-sectional study, we collected blood samples and clinical data from COVID-19 patients. The severity of COVID-19 was classified as mild (no hospital admission), severe (ward admission), and critical (ICU admission). ICU non-COVID-19 patients were also included and plasma from healthy age, sex, and BMI-matched individuals obtained from Lifelines. Multi-analyte profiling of plasma adipokines (Leptin, Adiponectin, Resistin, Visfatin) and inflammatory markers (IL-6, TNFα, IL-10) were determined using Luminex multiplex assays.

**Results:**

Between March and December 2020, 260 SARS-CoV-2 infected individuals (age: 65 [56–74] BMI 27.0 [24.4–30.6]) were included: 30 mild, 159 severe, and 71 critical patients. Circulating leptin levels were reduced in critically ill patients with a high BMI yet this decrease was absent in patients that were administered dexamethasone. Visfatin levels were higher in critical COVID-19 patients compared to non-COVID-ICU, mild and severe patients (4.7 vs 3.4, 3.0, and 3.72 ng/mL respectively, *p* < 0.05). Lower Adiponectin levels, but higher Resistin levels were found in severe and critical patients, compared to those that did not require hospitalization (3.65, 2.7 vs 7.9 µg/mL, *p* < 0.001, and 18.2, 22.0 vs 11.0 ng/mL *p* < 0.001).

**Conclusion:**

Circulating adipokine levels are associated with COVID-19 hospitalization, i.e., the need for oxygen support (general ward), or the need for mechanical ventilation and other organ support in the ICU, but not mortality.

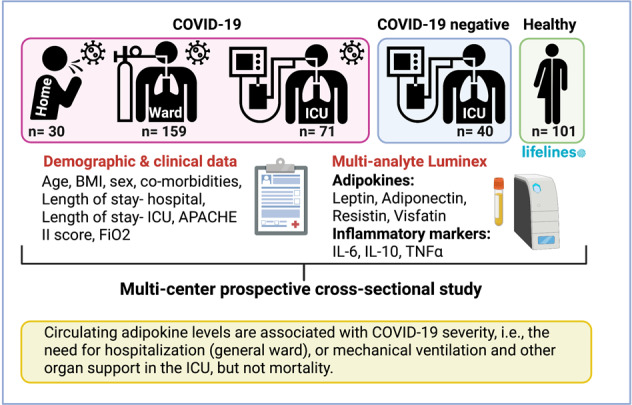

## Introduction

Infection with severe acute respiratory syndrome coronavirus 2 (SARS-CoV-2) causing coronavirus disease 2019 (COVID-19) is a multisystemic disease that can result in a range of clinical symptoms; from mild to severe pneumonia with acute respiratory distress syndrome (ARDS) [1–3]. Obesity has been shown to be a major risk factor for severe COVID-19, the need for organ support in the intensive care unit (ICU), and mortality [4–7]. More than 70% of the adults with COVID-19 admitted to the ICU are overweight or obese [8]. This might imply that excess and/or dysfunctional adipose tissue plays an important role in regulating systemic and pulmonary inflammatory responses against infection, which lead to excessive lung injury and respiratory failure. However, the precise underlying mechanisms are still poorly understood. Individuals with obesity do not only have excessive adipose tissue accumulation but also a dysregulated secretion of inflammatory adipokines and cytokines by adipose tissue which can not only affect the metabolism of tissues and organs but can also cause a chronic pro-inflammatory state predisposing them to thrombosis and other endothelial disturbances [9, 10]. We and others have hypothesized that this chronic inflammatory state in individuals with obesity might exacerbate the immunopathology associated with SARS-CoV-2 infection in patients with obesity thereby rendering them susceptible to severe organ injury [11, 12]. In line with this hypothesis, recent small studies from our group and others identified that plasma leptin was increased in COVID-19 patients admitted to the ICU [11, 13, 14]. Yet a decrease in Leptin in severe COVID-19 patients compared to mild and moderate COVID-19 was also found [15].

We designed a multicentre study comparing mild, severe and critically ill patients to investigate whether plasma adipokine levels were associated with COVID-19 severity, systemic inflammation, clinical parameters, and the outcome of SARS-CoV-2 infected patients. To date, this is the largest study to investigate the relationship between multiple circulating adipokines and COVID-19 severity and outcome.

## Methods

### Study design

In this multi-centre prospective cross-sectional study, we collected blood samples and clinical data from COVID-19 patients admitted to the University Medical Center Groningen (UMCG) and Amphia hospital in the Netherlands. The inclusion periods were 6th of March 2020 to 3rd of April 2020 (Amphia hospital; 1st wave), 24th of April to 6th of June 2020 (UMCG; 1st wave) and 28th of September 2020 to 3rd of December 2020 (UMCG; 2nd wave). For this study we defined the severity of COVID-19 on whether hospitalization to the ward or ICU was needed. The participants were categorised into the following 3 groups: mild (no hospital admission), severe (hospital admission to the general ward), and critical COVID-19 (intensive care unit (ICU) admission). Multi-analyte profiling of plasma adipokines (Leptin, Adiponectin, Resistin and Visfatin) and inflammatory markers (IL-6, TNFα, IL-10) were determined and compared to samples obtained from non-COVID-19 ICU patients, and age, sex, and BMI-matched healthy control individuals.

### Patient and participant selection

#### Severe and critical COVID-19 patients

All adult patients admitted to the UMCG and Amphia hospital diagnosed with COVID-19 during the initial 2 COVID-19 waves in the Netherlands were included in this study. SARS-CoV-2 infection was confirmed by RT-PCR of oropharyngeal or nasopharyngeal swabs. Patients were treated according to the local COVID-19 treatment protocol. Routine ICU management included selective digestive tract decontamination and high dose anticoagulation with low-molecular-weight heparin (LWMH) (87IE/kg twice daily). During the first COVID-19 wave, routine ward and ICU treatment initially included Chloroquine until the Dutch National Institute for Public Health and Environment advised against its use at the end of March 2020. All patients admitted to the hospital from July 2020 onwards (2nd wave) received Dexamethasone 6 mg daily if they needed additional oxygen therapy and some patients also received Remdesivir if the symptom duration was less than 10 days. We excluded patients who died in the general ward. These patients had an ICU indication but due to treatment restrictions were not admitted to the ICU. We contemplated including these patients in the critical COVID-19 group, but since they were not admitted to the ICU, were not mechanically ventilated etc, they are still a distinct group of patients compared to the critical COVID-19 patient group that we describe in this study. For this reason, we excluded these patients. None of the hospitalized COVID-19 patients were vaccinated against SARS-CoV-2. The need for informed consent was waived (UMCG METc 2020/492, and Medical Research Ethics Committees United W20.248/Central Research Committee Amphia N2020-0380) as the analyses were performed using residual plasma samples drawn during routine patient care.

### Mild COVID-19 patients

To compare severe and critically ill patients to SARS-CoV −2 infected individuals who developed mild or no symptoms, we used serum samples and demographic data from 30 individuals that were part of the COVID-HOME study [16]. This is an ongoing prospective observational cohort study carried out in the Northern part of the Netherlands aimed at gaining insights into COVID-19 in subjects who were not hospitalized. SARS-CoV-2 infection was confirmed in all individuals by RT-PCR of nasopharyngeal swabs. All individuals were unvaccinated against SARS-CoV-2 at inclusion. The COVID-HOME study was approved by the METc of the UMCG (METc 2020/158). All subjects provided written informed consent for the use of their data and biomaterials for research purposes.

### Critical non-COVID-19 patients

Plasma samples and clinical data were collected from 40 SARS-CoV-2-negative critically ill patients acutely admitted to the ICU-UMCG between the 24th of April and 6th of June 2020 (UMCG; 1st wave). All patients were confirmed to be SARS-CoV-2-negative and were admitted to the ICU for various reasons which included sepsis, respiratory insufficiency, cardiac arrest, trauma, and acute neurological illness. Elective surgical patients admitted to the ICU-UMCG during the same period were excluded from this study. The need for informed consent was waived as the analyses were performed using residual plasma samples drawn during routine patient care. (UMCG METc 2020/492).

### Healthy control individuals

We obtained plasma from 101 healthy individuals participating in the Lifelines Cohort Study that were age, sex and BMI-matched to our ICU population. All samples were collected before the COVID-19 pandemic and are therefore from SARS-CoV-2-negative individuals. Lifelines is a multi-disciplinary prospective population-based cohort study examining in a unique three-generation design the health and health-related behaviours of 167.729 persons living in the North of the Netherlands. Additional information about the Lifelines Cohort is available elsewhere [17].

### Multi-analyte analysis using Luminex

Heparinized blood was centrifuged (1300 G for 15 min at 4 °C) and the resulting plasma was collected for all hospitalized patients. Serum was collected from mildly symptomatic individuals. Samples were stored at −80 °C until analysis. For the Luminex analysis, all hospitalized patients that had a blood sample taken within 48 h after admission to the general ward and within 72 h after ICU admission were included. In the non-hospitalized mild COVID-19 cohort, blood samples were taken 7–9 days after testing positive for COVID-19. The reasoning for choosing this later timepoint is related to the day of COVID-19 patient hospitalization. Severe and critical COVID-19 patients included in our cohort were admitted to either the general ward or ICU around 8–10 days after initial COVID-19 symptoms (Supplemental Fig. 1). By doing so, we aimed to align all groups for comparison purposes as best as possible. Multianalyte profiling of samples was performed using custom-made Human Luminex xMAP multiplex assays (R&D Systems, Abingdon, UK) according to the manufacturers’ instructions and read on a Luminex 200 instrument (Luminex, Austen, TX, USA). Data analysis was performed using the xPONENT v4.2 software (Luminex). For this analysis, the adipokines: leptin, adiponectin, resistin, and visfatin, as well as cytokines: TNF-α, IL-6, and IL-10 levels were determined. Values that were below the lower limit of detection were set to the lowest value that we could accurately determine; if the upper limit of detection was reached, these values were set to the upper limit of quantification. Inter-assay variation was monitored using 3 of the 7 calibration line points made on the first day of measurement, aliquoted and stored at −80 °C. These 3 aliquoted samples were thawed and used as internal controls each day the analysis was performed. For each analyte the CV’s were Leptin 6.7%, Adiponectin 11%, Resistin 17.4%, Visfatin 31.9%, IL-6 10.2%, IL-10 7.3% and TNFα 14%.

### Data collection

Patient clinical and demographic information was extracted from electronic medical records in the case of hospitalized individuals. Data from mild patients of the COVID HOME study was collected through Case Report Forms (CRFs) applied to the study participants during their acute disease. Clinical information consisted of age, sex, body mass index (BMI), medical history and clinical course. Data was captured in a modified version of the World Health Organisation (WHO) electronic Case Report Form (eCRF) [18] in the clinical database infrastructure Research Electronic Data Capture (REDCap, Vanderbilt University, USA) [19]. BMI was used to estimate body fat mass and is defined as a person’s weight in kilograms divided by the square of height in meters (kg/m^2^). The extent of obesity was defined according to the WHO classification: BMI 18.8–24.9 kg/m^2^ = normal weight, 25–29.9 kg/m^2^ = overweight, ≥30.0 kg/m^2^ = obese. The BMI distribution of included subjects separated by severity; mild (home), severe (ward), and critical (ICU) can be found in Supplemental Fig. 2.

### Statistical analysis

Continuous variables are expressed as median with inter-quartile range [IQR]. Categorical data are expressed as frequencies and percentages. The Shapiro–Wilk test was used to determine if the data was normally distributed. Comparisons between the different groups were tested using the Kruskal–Wallis test, Wilcoxon-Mann-Whitney test for median and IQR and χ^2^ or Fisher’s exact test for percentages. Correlations were determined using Spearman’s correlation, adjusting for multiple comparisons using Bonferroni correction. Analyses were performed using SPSS® version 25 (IBM, Chicago, IL, USA) and GraphPad Prism Software v9 (La Jolla, CA, USA). Differences were considered statistically significant when the *p*-value < 0.05.

## Results

### Baseline characteristics of patient groups

**Fig. 1:**
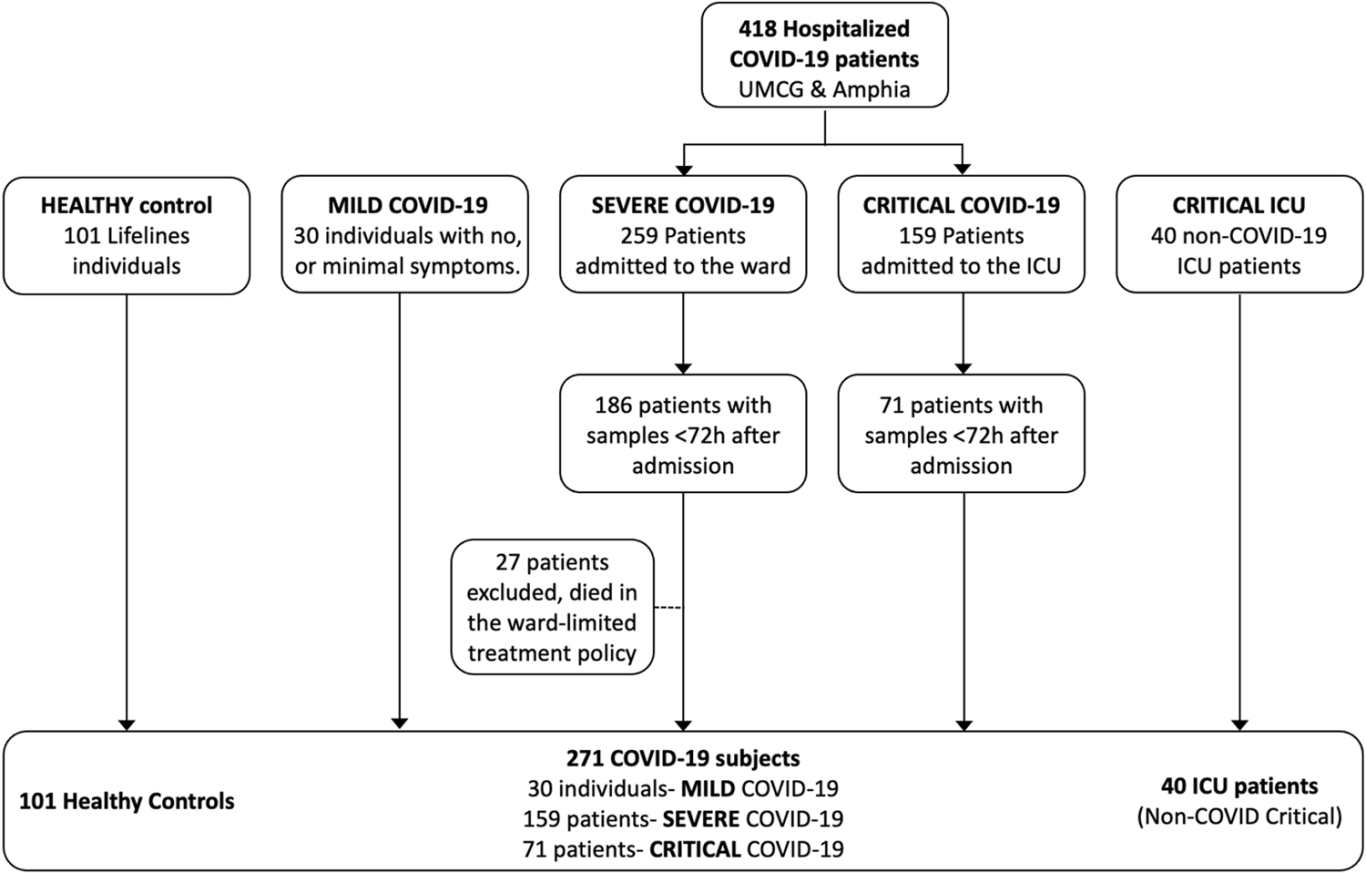
Study cohorts. Overview of the cohorts and patient selection included in our study.

From the 418 COVID-19 patients admitted to the UMCG and Amphia hospitals, 186 (72%) ward patients had plasma samples available within 48 h of admission and were included in the severe COVID-19 group while 71 (45%) patients had a plasma sample within 72 h after admission to the ICU and were included in the critical COVID-19 group. Twenty-seven hospitalized COVID-19 patients that required ICU care but were not admitted to the ICU due to limited treatment policy were excluded from this study. Additionally, we included serum from 30 SARS-CoV-2-infected individuals with mild symptoms (Mild COVID-19), and plasma from 40 SARS-CoV-2-negative patients acutely admitted to the ICU-UMCG (critical non-COVID). For the analysis we additionally included samples from 101 healthy controls that were age, sex and BMI-matched to the critical COVID-19 patients that were admitted to the ICU during the first wave of the pandemic. Healthy individuals were however older than mild patients (*p* = 0.031) and consisted of more males compared to the mild and severe COVID-19 patients (*p* = 0.002 and *p* = 0.006 respectively). In total 271 SARS-CoV-2-infected subjects were included for analysis (Fig. 1).

Patient characteristics, complications, and outcome are described in Table 1. The median age of critical COVID-19 patients was 66 years and comparable to critical non-COVID-19 patients (65 years). Almost 70% of critical COVID-19 patients were male which was comparable to non-COVID-19 critical patients (75%). From the COVID-19 patients admitted to the general ward 54% were male. The BMI of severe and critical COVID-19 patients were similar 27 and 28 kg/m^2^ respectively and did not differ from critical non-COVID-19 patients. As expected, higher complication rates were associated with disease severity, critical COVID-19 patients experienced more thromboembolic events (pulmonary embolism [25.4% vs 3.1%], stroke [9.9% vs 0.6%], acute kidney injury [49.3% vs 6.9%] and liver dysfunction [47.9% vs 15.1%] compared to severe COVID-19 patients. The median duration of hospital stay was 5 [4–9] days for severe COVID-19 patients, and 17 [11–25] days for critical COVID-19 patients (*p* < 0.001). Although the total number of hospital days between COVID-19 and non-COVID-19 critical patients did not differ, the ICU length of stay was longer for critical COVID-19 patients compared to non-COVID critical patients, 10 versus 7 days respectively (*p* = 0.001). Mortality of critical COVID-19 patients was comparable to non-COVID-19 critical patients [43.7% vs 42.5%].

**Table 1:** Baseline patients’ clinical characteristics, comorbidities, therapies, and outcome.

Variable	Mild COVID-19 (A)	Severe COVID-19 (B)	Critical COVID-19 (C)	Critical non-COVID (D)	*P* value A vs B	*P* value A vs C	*P* value B vs C	*P* value C vs D
	Home *n* = 30	Ward *n* = 159	ICU *n* = 71	ICU *n* = 40				
*Demographics*								
Healthy controls (*n* = 101): age (years)= 64 [57–73], Male, *n* (%)=72 (71.3%), BMI (kg/m2)= 28 [26–31]								
Age (years)	60 [53–64]	67 [56–76]	66 [58–72]	65 [50–74]	0.004	0.004	0.628	0.214
Male, *n* (%)	12 (40.0)	86 (54.1)	48 (67.6)	30 (75.0)	0.157	0.010	0.055	0.413
BMI (kg/m^2^)	26.0 [23–29]	27 [24–31]	28 [25–31]	27 [25–29]	0.171	0.039	0.335	0.229
*Comorbidities*								
Pulmonary disease, *n* (%)	4 (13.3)	35 (22.0)	18 (25.4)	7 (17.5)	0.315	0.205	0.578	0.342
Chronic cardiac disease, *n* (%)	2 (6.7)	40 (25.2)	21 (29.6)	17 (42.5)	0.030	0.014	0.483	0.168
Chronic kidney disease, *n* (%)	0 (0)	18 (11.3)	4 (5.6)	6 (15.0)	0.081	0.320	0.176	0.164
Hypertension, *n* (%)	4 (13.3)	55 (34.6)	25 (35.2)	12 (30.0)	0.026	0.032	0.927	0.576
Diabetes, *n* (%)	2 (6.7)	35 (22.0)	11 (15.5)	3 (7.5)	0.060	0.336	0.253	0.223
*Clinical course*								
O2 hospital admission (L/min)	-	3 [0–5]	8 [4–17]				<0.001	
Hospital length of stay, days	-	5 [4–9]	17 [11–25]	18 [9–29]			<0.001	0.786
*Treatment*	-							
Antiviral	-	35 (22.0)	10 (14.1)				0.161	
Antibiotics	-	99 (62.3)	71 (100)				<0.001	
Corticosteroids	-	54 (34.0)	31 (43.7)				0.159	
Antifungal therapy	-	0 (0)	17 (23.9)				<0.001	
Chloroquine	-	73 (45.9)	39 (54.9)				0.206	
*ICU characteristics*								
APACHE II score			16 [13–20]	23 [21–28]				<0.001
ICU length of stay, days			10 [7–22]	7 [4–13]				0.001
Duration MV, days			10 [6–22]	5 [3–9]				<0.001
PaO2/FiO2 after intubation			131 [99–187]	188 [143–372]				<0.001
Prone ventilation			47 (69.1)	0 (0)				<0.001
Tracheostomy			6 (8.8)	3 (7.5)				0.999
ECLS			2 (2.9)	1 (2.5)				0.999
*Complications*								
Pulmonary embolism	-	5 (3.1)	18 (25.4)	0 (0)			<0.001	0.001
Pneumothorax	-	1 (0.6)	2 (2.8)	2 (5.0)			0.226	0.618
Stroke	-	1 (0.6)	7 (9.9)	1 (2.5)			<0.001	0.255
Cardiac ischemia	-	5 (3.1)	4 (5.6)	0 (0)			0.463	0.295
Bacteremia^*^	-	0 (0)	9 (12.7)	3 (7.5)			<0.001	0.532
Acute kidney injury^**^	-	11 (6.9)	35 (49.3)	9 (22.5)			<0.001	0.006
Renal replacement therapy		0 (0)	13 (18.3)	7 (17.5)			<0.001	0.915
Liver dysfunction^***^	-	24 (15.1)	34 (47.9)	26 [65]			<0.001	0.082
*Outcome*								
Death			31 (43.7)	17 (42.5)				0.906

Data is presented as median [iQR], or n and (percentage). *P*-values are calculated using, Kruskal–Wallis, Mann– Whitney U, Chi-squared test or Fishers Exact Test. APACHE Acute Physiology And Chronic Health Evaluation, *MV* Mechanical Ventilation, *BMI* body mass index, *MV* Mechanical Ventilation, *ECLS* Extra Corporeal Life Support, *ICU* Intensive Care Unit, ^*^positive blood cultures, ^**^increase in serum creatinine by 26.5 umol/L within 48 h, or >1,5 times baseline within the prior 7 days, ^***^an increase in blood bilirubin, alanine transaminase or aspartate transaminase that is twice the upper limit of the normal range.

### Plasma adipokine and cytokine levels are associated with COVID-19 severity

Our previous study found that plasma leptin levels were increased in critical COVID-19 patients compared to non-COVID-19 critical patients [11]. To validate these findings, and to investigate the potential role of other adipokines this study was performed. Plasma adipokine levels are known to be influenced by the extent of adiposity and age [20]. Notably, most severe, and critical COVID-19 patients are overweight or obese (Supplemental Fig. 2). In this study the age and BMI of severe and critical COVID-19 patients, non-COVID-19 critical patients and healthy control groups were all similar (Table 1).

**Fig. 2:**
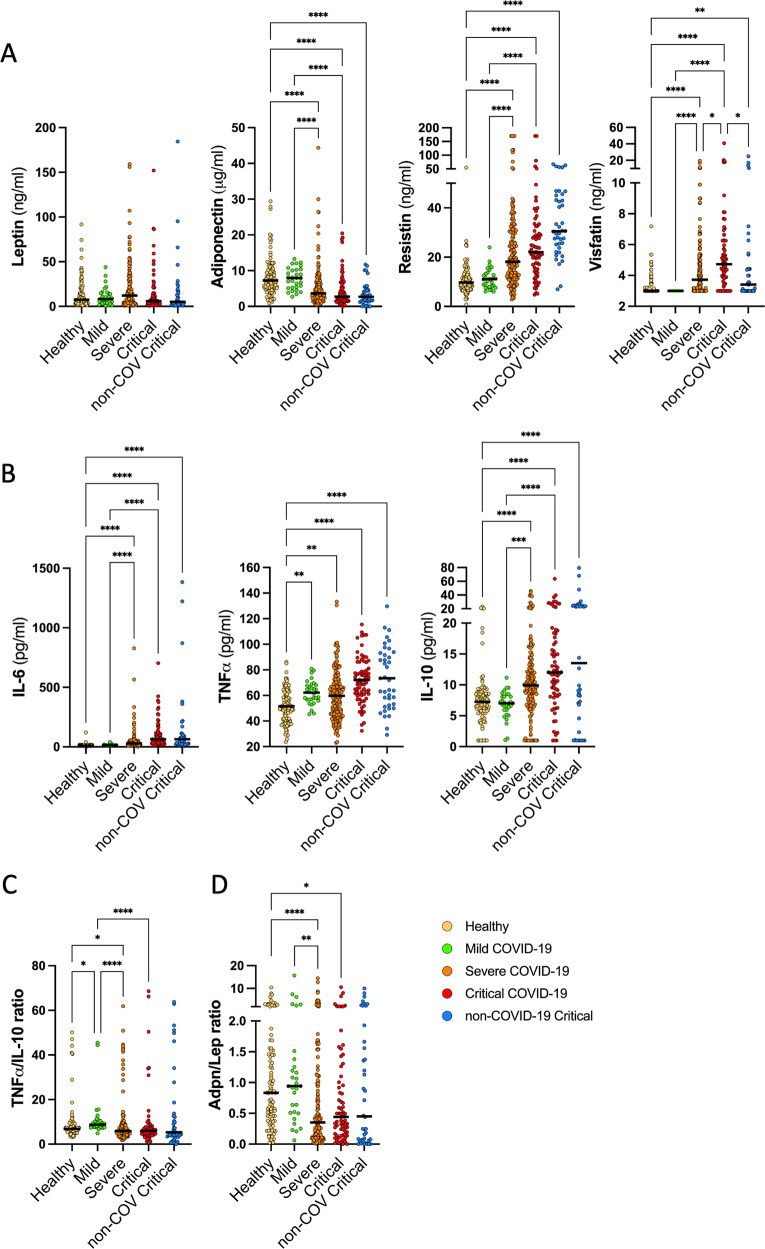
Circulating adipokine and cytokine levels between the different groups. **A** Circulating levels of adipokines Leptin, Adiponectin, Resistin and Visfatin, and **B** cytokines IL-6, TNFα, and IL-10 were determined between the different groups. **C** The adiponectin:Leptin ratio, and **D** the TNFα:IL-10 ratio were also determined. Each symbol represents from a single individual or patient. The black line represents the median. *P* values were calculated using the Kruskal–Wallis test with a post-hoc Mann Whitney U test. *p* < 0.05 were considered significant. *p < 0.05, *p* < 0.01, ***p < 0.001, ****p < 0.0001. In the absence of ‘*‘ the data is non-significant.

We found that leptin levels were similar between critical COVID-19 and non-COVID-19 critical patients. Moreover, we did not find an association between leptin levels and COVID-19 severity (Fig. 2A). Leptin levels are known to be associated with BMI. Unfortunately, our cohort size was too small to stratify for BMI categories so instead we proceeded to stratify patients based on the median BMI of 27.2 kg/m2. The Low BMI group had a BMI < 27.2 kg/m2 and the high BMI group had a BMI > 27.2 kg/m2 (Supplemental Table 1). Interestingly, severe COVID-19 patients with a high BMI had higher levels of plasma leptin than critical COVID-19 patients with a similarly high BMI, respectively 16.2 ng/ml vs 6.3 ng/ml, *p* = 0.017. Implying that Leptin levels are reduced in critically ill COVID-19 patients who have a high BMI. Adiponectin levels were significantly reduced to similar levels in both severe and critical COVID-19 patients but were also reduced in non-COVID critical patients, compared to mild COVID-19 and healthy controls (Fig. 2A). The Adiponectin to Leptin (Adpn/Lep) ratio, a marker of adipose tissue dysfunction, was similarly reduced in all hospitalized COVID-19 and non-COVID critical patients compared to mild COVID-19 and healthy controls (Fig. 2D). Individuals with mild COVID-19 had similar adiponectin levels and Adpn/Lep ratio to healthy control individuals (Fig. 2A, C). Resistin levels were increased to the same extent in both severe and critical COVID-19 patients as well as non-COVID critical patients (Fig. 2A). Visfatin levels were all below the lower limit of detection in individuals with mild COVID-19 as well as a subpopulation of patients in the other groups (Fig. 2A). Visfatin levels were higher in critical COVID-19 patients compared to severe COVID-19 patients. Moreover, this increase was specific to critical COVID-19 patients since non-COVID-19 critical patients had significantly lower levels of Visfatin (Fig. 2A).

**Fig. 3:**
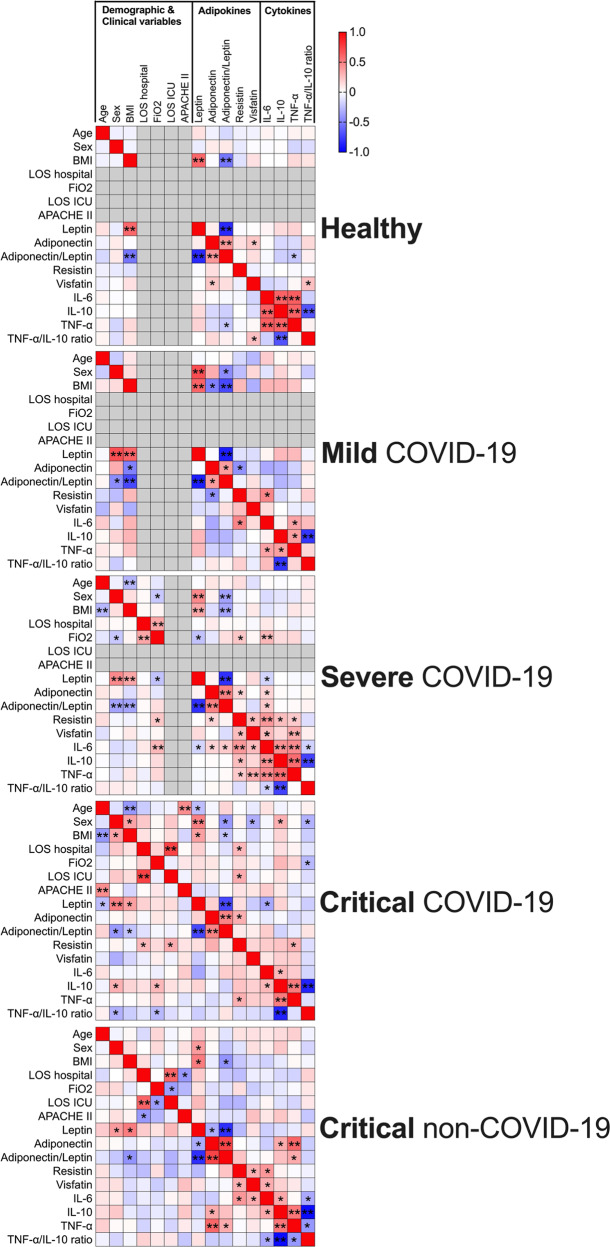
Correlation of plasma adipokine levels and clinical parameters. Heatmaps of the Spearman correlation (r) between adipokines, cytokines, age, sex, BMI, and clinical variables. “*” represents significant correlations, *p* < 0.05. “**” represents significant correlations after adjusting for multiple testing with Bonferroni correction.

The inflammatory response to SARS-CoV-2 plays an important role in the progression to severe COVID-19 disease. To investigate whether there was an association between adipokine levels and pro- and anti-inflammatory responses, we also determined the plasma levels of pro-inflammatory markers IL-6 and TNFα, and anti-inflammatory cytokine IL-10. The cytokines, which we determined appear to be associated with COVID-19 severity, yet no statistical difference was found between severe and critical COVID-19 patients (Fig. 2B). TNFα but not IL-6 or IL-10 levels were increased in mild COVID-19 patients, compared to healthy controls. IL-6, TNFα, and IL-10 levels were highest in severe and critical COVID-19 patients, with comparable levels to non-COVID-19 critical patients. The TNFα/IL-10 radio, an indication of the balance between key pro- to anti-inflammatory levels, was also reduced to a similar level in severe, critical COVID-19, and non-COVID-19 critical patients (Fig. 2D). These findings suggest a more general inflammatory response associated with critical illness rather than a COVID-19-specific cytokine storm. In severe COVID-19 patients, IL-6 levels positively correlated with the levels of Adiponectin, Visfatin, Resistin and negatively correlated with plasma leptin levels. IL-10 and TNFα plasma levels also correlated with the levels of Resistin and Visfatin (Fig. 3). Although significant, all correlations were relatively weak. Moreover, most of these correlations were lost in critical COVID-19 patients except the negative correlation of IL-6 with leptin levels, and positive correlations found between IL-10 and TNFα and Resistin. In fact, the clearly different correlation matrixes based on COVID-19 severity (Fig. 3) suggest distinct systemic plasma profiles and concomitant pathophysiological processes.

### Plasma adipokine levels and clinical parameters of severe COVID-19 disease

We proceeded to investigate whether plasma adipokine or cytokine levels were associated with specific clinical features of hospitalized patients. In severe, but not critical COVID-19 patients, we found that plasma Leptin, Resistin and IL-6 correlated with the fraction of inspired oxygen (FiO2) but not the length of hospital stay (Fig. 3). In contrast, only Resistin levels correlated with the duration of hospital and ICU stay in critical COVID-19 patients. However, after Bonferroni correction for multiple testing, these parameters did not pass the adjusted threshold for significance. Additionally, the plasma levels of adipokines and cytokines were not associated with the severity of critical illness (APACHE II score) (Fig. 3).

### Plasma adipokine levels are not associated with the mortality of critical COVID-19 patients

Several studies have shown that obesity is associated with mortality of COVID-19 patients [4–7] potentially driven by dysregulated production and systemic levels of adipokines and cytokines [9, 21]. We therefore investigated the association between plasma adipokines and COVID-19 severity, systemic inflammation, clinical parameters, and the outcome of COVID-19 patients. The mortality rate of COVID-19 patients was 43.7% (*n* = 31/71). Critical COVID-19 non-survivors were older (71 versus 62 years old) (Table 2). The percentage of males, the patient BMI and APACHE II score were similar between both the survivors and non-survivors. Plasma levels of all determined adipokines were similar between both groups indicating that altered adipokine levels were not associated with mortality in critical COVID-19 patients. Increased levels of IL-6 were observed in COVID-19 non-survivors; however, this was not significant.

**Table 2:** Plasma adipokine levels are not associated with mortality of critically ill COVID-19 patients.

Variable	Critical COVID-19 Survivors *n* = 40	Critical COVID-19 Non-survivors *n* = 31	*p* value
*Demographics*			
Age (years)	62.5 [56.3–69]	71 [63–75]	0.005
Male, *n* (%)	27 (67.5)	21 (67.7)	0.983
BMI (kg/m^2^)	27.8 [24.9–30.7]	27.3 [25.2–30.8]	0.954
Total hospital days	18 [13–38]	16 [10–24]	0.182
*ICU Characteristics*			
Total ICU days	10 [7–23]	13 [7–22]	0.995
Total ventilated days	8 [5–22]	13 [7–22]	0.389
PaO2/FiO2 after intubation	131 [99–202]	127 [98–152]	0.589
APACHE II score	16 [11–20]	17 [15–23]	0.120
Prone ventilation, *n* (%)	22 (59.5)	25 (80.6)	0.060
Tracheostomy, *n* (%)	5 (12.5)	1 (3.2)	0.136
Renal Replacement Therapy, *n* (%)	5 (12.5)	8 (25.8)	0.150
ECLS, *n* (%)	0 (0)	2 (6.5)	0.204
Adipokine/Cytokines			
Leptin, ng/mL	6.0 [3.0–14–9]	5.9 [2.3–14.7]	0.615
Adiponectin, µg/mL	2.4 [1.5–4.2]	3.0 [1.7–6.9]	0.279
Adiponectin/Leptin ratio	0.49 [0.14–1.00]	0.34 [0.20–1.26]	0.718
Resistin, ng/mL	21 [14–33]	24 [18–28]	0.504
Visfatin, ng/mL	4.5 [3.5–6.3]	4.7 [3.6–6.3]	0.995
IL-6, pg/mL	53 [33–108]	75 [43–128]	0.259
IL-10, pg/mL	12.2 [8.6–19.2]	11.8 [8.0–15.6]	0.356
TNF-α, pg/mL	70 [60–86]	72 [63–81]	0.615
TNF-α/IL-10 ratio	5.9 [4.6–7.3]	6.6 [4.6–9.3]	0.301

Data is presented as median [iQR], or n and percentage. *P*-values are calculated using Mann–Whitney U, Chi-squared test or Fishers Exact Test. *APACHE* Acute Physiology And Chronic Health Evaluation, *BMI* body mass index, *ECLS* Extra Corporeal Life Support, *ICU* Intensive Care Unit.

### Comparison of adipokine and cytokine levels from hospitalized patients included in the first and second waves of the COVID-19 pandemic

In this study we collected plasma from hospitalized patients during the first wave and second wave of infections. The management and treatment protocol of hospitalized patients admitted during the second wave was adjusted based on the results from the RECOVERY and REMAP-CAP randomized controlled clinical trials that demonstrated a meaningful mortality benefit in patients receiving either dexamethasone or IL-6 inhibitors [22, 23]. Dexamethasone and/or IL-6 receptor inhibitor Tocilizumab were subsequently swiftly implemented as standard clinical protocol. In our cohort, 100% critical COVID-19 patients and 85% severe COVID-19 patients admitted during the second wave of infections received corticosteroids compared to 9% during the first wave (Table 3). Only 3 patients (1 in the first wave, and 2 in the second wave) received Tocilizumab in combination with corticosteroids.

**Table 3:** Comparison between the 1st and 2nd wave of COVID-19 admissions to the ward and ICU.

Variable	Severe COVID-19 Ward (A) 1st wave	Severe COVID-19 Ward (B) 2nd wave	Critical COVID-19 ICU (C) 1st wave	Critical COVID-19 ICU (D) 2nd wave	*P* value A vs B	*P* value C vs D
	*n* = 107	*n* = 52	*n* = 44	*n* = 27		
*Demographics*						
Age (years)	68 [57–77]	65 [54.5–72.8]	68 [58–74]	66 [56–72]	0.146	0.374
Male, *n* (%)	59 (55.1)	27 (51.9)	30 (68.2)	18 (66.7)	0.703	0.895
BMI (kg/m^2^)	26.6 [23.7–30.3]	28.1 [24.8–30.9]	27.8 [24.8–30.7]	27.0 [25.2–32.3]	0.314	0.873
Comorbidities						
Pulmonary disease, *n* (%)	22 (20.6)	13 (25.0)	10 (22.7)	8 (29.6)	0.526	0.516
Chronic cardiac disease, *n* (%)	30 (28.0)	10 (19.2)	11 (25.0)	10 (37.0)	0.230	0.281
Chronic kidney disease, *n* (%)	12 (11.2)	6 (11.5)	2 (4.5)	2 (7.4)	0.952	0.632
Hypertension, *n* (%)	36 (33.6)	19 (36.5)	15 (34.1)	10 (37.0)	0.719	0.801
Diabetes, *n* (%)	24 (22.4)	11 (21.2)	6 (13.6)	5 (18.5)	0.855	0.737
*Clinical course*						
O2 hospital admission (L/min)	2 [0–4]	2 [0–4]	12 [3.3–15]	4 [3–13.8]	0.747	0.181
Hospital length of stay, days	5 [4–9]	5 [4–8]	16 [8–28.8]	18 [16–24]	0.777	0.092
*Treatment*						
Antiviral	1 (0.9)	34 (65.4)	6 (13.6)	4 (14.8)	<0.001	1.000
Antibiotics	82 (76.6)	17 (32.7)	44 (100)	27 (100)	<0.001	1.000
Corticosteroids	10 (9.3)	44 (84.6)	4 (9.1)	27 (100)	<0.001	<0.001
Antifungal therapy	0 (0)	0 (0)	13 (29.5)	4 (14.8)	1.000	0.158
Chloroquine	73 (68.2)	0 (0)	39 (88.6)	0 (0)	<0.001	<0.001
ICU Characteristics						
APACHE II score			15.5 [12.0–23.3]	16 [13.8–20.0]		0.909
ICU length of stay, days			10 [6.8–25.8]	12 [7–21]		0.786
Duration of MV, days			9.5 [6.0–22.8]	10.5 [5.3–20.5]		0.699
PaO2/FiO2 after intubation			126 [99–181]	148 [95–193]		0.668
Prone ventilation			34 (77.3)	13 (54.2)		0.049
Tracheostomy			6 (13.6)	0 (0)		0.083
ECLS			2 (4.5)	0 (0)		0.536
*Complications*						
Pulmonary embolism	3 (2.8)	2 (3.8)	13 (29.5)	5 (18.5)	0.662	0.300
Pneumothorax	1 (0.9)	0 (0)	1 (2.3)	1 (3.7)	1.000	1.000
Stroke	0 (0)	1 (1.9)	6 (13.6)	1 (3.7)	0.327	0.240
Cardiac arrhythmia	4 (3.7)	2 (3.8)	10 (22.7)	6 (22.2)	1.000	0.961
Cardiac ischemia	4 (3.7)	1 (1.9)	3 (6.8)	1 (3.7)	1.000	1.000
Bacteremia^*^			6 (13.6)	3 (11.1)		1.000
Acute kidney injury^**^	6 (5.6)	5 (9.6)	26 (59.1)	9 (33.3)	0.341	0.035
Renal replacement therapy			12 (27.3)	1 (3.7)		0.013
Liver dysfunction^***^	18 (16.8)	6 (11.5)	21 (47.7)	13 (48.1)	0.383	0.973
*Outcome*						
Death			22 (50.0)	9 (33.3)		0.169
*Adipokine/Cytokines*						
Leptin, ng/mL	8.4 [3.3–16.9]	17.4 [7.9–40.0]	4.0 [1.9–9.6]	9.5 [5.9–51.0]	<0.001	<0.001
Adiponectin, µg/mL	3.5 [2.2–6.4]	4.2 [2.5–6.7]	2.4 [1.7–4.1]	3.9 [1.5–7.1]	0.383	0.127
Adiponectin/Leptin ratio	0.4 [0.2–1.1]	0.2 [0.1–0.6]	0.5 [0.2–1.4]	0.3 [0.1–1.0]	0.009	0.133
Resistin, ng/mL	17.9 [12.2–26.5]	18.3 [10.7–37.9]	22.2 [14.8–28.4]	21.8 [17.5–38.7]	0.845	0.436
Visfatin, ng/mL	3.6 [3.0–4.8]	4.0 [3.0–5.6]	4.7 [3.6–6.6]	4.7 [3.5–5.7]	0.130	0.703
IL-6, pg/mL	27.9 [21.1–40.2]	33.7 [19.9–55.3]	92.2 [55.3–138.2]	41.3 [27.2–51.2]	0.171	<0.001
IL-10, pg/mL	9.6 [6.8–12.4]	10.3 [8.1–13.6]	11.8 [8.3–18.4]	12.0 [7.0–14.9]	0.157	0.511
TNF-α, pg/mL	51.2 [42.3–65.1]	66.9 [57.4–81.9]	69.3 [57.5–79.5]	75.9 [62.6–86.7]	<0.001	0.314
TNF-α/IL-10 ratio	5.6 [4.1–7.9]	6.4 [4.8–8.0]	5.3 [4.0–7.7]	6.6 [5.2–7.4]	0.241	0.121

First COVID-19 wave patient were admitted to the general ward (A) and ICU (C) between March and July 2020, Second Wave COVID-19 patients were admitted to the general ward (B) and ICU (D) between September and December 2020. Data is presented as median [iQR], or n and percentage. P-values are calculated using Mann–Whitney U, Chi-squared test or Fishers Exact Test. *APACHE* Acute Physiology And Chronic Health Evaluation, *BMI* body mass index, *MV* Mechanical Ventilation, *ECLS* Extra Corporeal Life Support, *ICU* Intensive Care Unit, ^*^positive blood cultures, ^**^increase in serum creatinine by 26.5 umol/L within 48 h, or >1,5 times baseline within the prior 7 days, ^***^an increase in blood bilirubin, alanine transaminase or aspartate transaminase that is twice the upper limit of the normal range.

The age, BMI, percentage of males and comorbidities did not differ between patients admitted to either the ward or ICU during the first and second COVID-19 waves (Table 3). The length of both hospital and ICU stay were similar between the first and second waves of severe and critical COVID-19 patients. Hospital mortality did not significantly differ between the first and second wave patients despite a lower incidence of acute kidney injury in second wave critical COVID-19 patients [33% 2nd wave versus 50% 1st wave]. Leptin levels were higher in the plasma from severe and critical COVID-19 patients during the second wave, [8.4; IQR 3.3–16.9] compared to the first wave [17.4; IQR 7.9–40.0] for severe, and [9.5; IQR 5.9–51.0] compared to [4.0; IQR 1.9–9.6] for critical COVID-19 patients. Moreover, IL-6 levels were reduced by 55% in critical COVID-19 patients from the second wave compared to the first wave [41.3; IQR 27.2–51.2 versus 92.2; IQR 55.3–138.2] but this was not observed for severe COVID-19 patients (Table 3). Adiponectin, Resistin, Visfatin, TNFα and IL-10 levels did not differ between the 2 COVID-19 waves of infection suggesting minimal influence of dexamethasone treatment.

## Discussion

Obesity is increasingly recognized as a risk factor for the progression to severe forms of COVID-19 [4–7] yet the mechanisms involved are still unclear. In this multicenter study examining a large cohort of SARS-CoV-2-infected individuals with mild to critical COVID-19 disease, we demonstrate that circulating adipokine and inflammatory cytokine levels are associated with COVID-19 severity, i.e., the need for hospitalization (general ward), or the need for mechanical ventilation and other organ support in the ICU, but not mortality.

In healthy individuals, there is a strong correlation between obesity and adipokines [24–27]. However, in the current study there was no correlation between BMI and the measured adipokines, except for leptin. A possible explanation for our findings could be that COVID-19 causes adipocyte dysfunction either by direct infection of SARS-CoV-2 in cells within the adipose tissue such as adipocytes, and/or through other indirect mechanisms. Currently, data demonstrating SARS-CoV-2 infection of adipose tissue in vivo in COVID-19 patients is scarce but has been reported in some postmortem patients [28–30]. In addition, studies have reported adipose tissue infection with SARS-CoV-2 in hamster [31] and macaque [32] in vivo models. In vitro studies have also shown that primary human adipocytes isolated from breast tissue were permissive to SARS-CoV-2 infection and that this resulted in reduced Adiponectin mRNA expression [31]. Hence, based on the evidence so far it is increasingly likely that direct infection can induce adipose tissue dysfunction and altered secretion of adipokines and cytokines, but direct evidence is still lacking.

Recent studies identified that plasma leptin was increased in COVID-19 patients admitted to the ICU [11, 13, 14], whereas a decrease in Leptin in severe COVID-19 patients compared to mild and moderate COVID-19 has also been reported [15]. It is well known that both leptin and adiponectin are associated with age, sex, and BMI and not all previous studies adjusted for these variables, nor did they include matched healthy control individuals. Therefore, a strength of our study was that we controlled for age, sex, and BMI for the allowing comparison of adipokine levels and COVID-19 severity. When we stratified patients based on BMI (high or low) we found plasma leptin levels were reduced in critical COVID-19 compared to severe COVID-19 patients with similar BMI corroborating previous results by Di Filippo et al. (2021). However, we also identified that circulating leptin levels were increased by dexamethasone treatment. This is in line with previous in vivo experimental studies that found that dexamethasone was a powerful stimulator of leptin production [33–35]. As a result, the reduction in leptin was observed only in critical COVID-19 patients admitted to the ICU during the first COVID-19 wave (without dexamethasone). It is hypothesized that leptin and/or other systemic adipokines could contribute to the endothelial activation and dysfunction observed in severe COVID-19 patients with obesity [11, 12]. We did not identify a direct positive relationship between the levels of leptin and IL-6 in hospitalized COVID-19 patients. Several other studies also did not find leptin to be increased in inflammatory conditions in humans such as experimental endotoxemia, sepsis, and HIV infection [36–38], despite an increase in IL-6 levels. Therefore, leptin may act as an inflammatory mediator in some conditions but not in others such as COVID-19. In line with these in vivo findings, we recently demonstrated that incubating endothelial cells (which normally produce a lot of IL-6 upon inflammatory stimulus) with recombinant leptin, and other adipose tissue mediators, did not result in an inflammatory response, nor promote SARS-CoV-2 infection [39].

Adiponectin has anti-inflammatory properties and is reduced in individuals with obesity. Previous studies found that Adiponectin leads to inhibition of IL-6 expression by murine pulmonary endothelial cells as well as reduction of lung inflammation in murine ARDS models [40]. In human bronchial epithelium, adiponectin also dampened the inflammatory response among others by inhibiting IL-6, and low circulating adiponectin levels in individuals with obesity may therefore contribute to SARS-CoV-2 susceptibility and increase the severity of the infection [41]. Here we show that adiponectin is further reduced in the setting of severe and critical COVID-19 and critical illness in general. This also resulted in a lower Adiponectin/Leptin ratio which has been proposed as a marker for adipose tissue dysfunction [42].

Circulating resistin levels were increased in hospitalized COVID-19 patients and in non-COVID-19 critical patients which is in line with previous findings demonstrating increased levels in patients with sepsis and septic shock as well as critically ill patients without infection [43–45]. We found that Visfatin levels were significantly elevated in severe and critical COVID-19 patients compared to mild individuals. Moreover, Visfatin levels in critical COVID-19 were significantly higher compared to non-COVID-19 critical patients which suggest a specific role for Visfatin in critical COVID-19 rather than critical illness in general. Visfatin is produced in visceral adipose tissue and its expression and secretion are associated with obesity [46]. Inflammatory cytokines and lipopolysaccharide have been shown to influence Visfatin expression [47, 48]. Additionally, Visfatin levels were shown to be increased in acute lung inflammation and sepsis [45, 49, 50]. Visfatin has been shown to induce a proinflammatory state in bronchial epithelial cells [50] and can also induce endothelial dysfunction [51]. Recently, a humanized Visfatin-neutralizing antibody was shown to reduce the severity of lung injury by 50% in rat and porcine experimental models of ARDS. Moreover, respiratory compliance was improved, and lung water imbalances reduced [52]. These published findings suggest that Visfatin might be directly associated with lung injury and/or organ failure in critical COVID-19 [53]. However, considering the lack of significant correlation between Visfatin and clinical variables in our current study, further studies are required to establish whether Visfatin plays a role in ARDS regardless of aetiology, or whether it plays an important role specifically in COVID-19. Sustained elevation of both Visfatin and Resistin was associated with the severity of critical illness in patients with sepsis (APACHE II) [45]. We did not find a correlation between admission plasma adipokine levels and APACHE II in critical COVID-19 patients. Therefore, longitudinal studies investigating circulating Visfatin and Resistin in COVID-19 patients might shed more light on the role of adipokines and critical illness severity and patient outcome.

Most published studies investigating plasma adipokine levels and COVID-19 severity are limited by their small sample size of COVID-19 patients. A strength of this cross-sectional study was the inclusion of a large cohort of patients from 2 different medical centres. However, our study also has several limitations which need to be considered. Since blood samples and clinical information were collected during pandemic conditions, we were limited to using the patient BMI as a simple and quick way to estimate body fat mass, whereas the hip-to-waist ratio or abdominal CT scans may say more about the amount of visceral fat in relation to the rest of the body fat. Hip-to-waist measurements were unfortunately not performed during the pandemic and the chest CT scans made are not of sufficient resolution to accurately say something about the extent of visceral fat [54, 55]. Nevertheless, previous studies have reported an association between BMI and waist circumference in patients testing positive for COVID-19. Higher adiposity markers (BMI, waist circumference, waist-to-hip ratio, and waist-to-height ratio) are associated with a greater risk of COVID-19 mortality [56]. For the analyses, all samples were plasma except the mild COVID-19 patients, which was serum. Despite the evidence that the use of serum leads to higher adipokine, and cytokine levels compared to heparin plasma from the same individuals [57–64], we have included serum samples from mild COVID-19 patients for our analysis. The use of serum samples for the mild COVID-19 group, still resulted in significantly lower levels compared to levels measured in plasma from hospitalized COVID-19 patients. However, a direct comparison between mild COVID-19 and the hospitalized COVID-19 and control cohort should be interpreted with caution. Due to the inter-assay CV and the fact that several samples were below the detection limit for Visfatin, we are unable to make strong conclusions from the results. We would therefore recommend that future studies investigating the role of Visfatin should be evaluated using a more sensitive assay in the lower range. Although we strived to collect blood samples at admission, that was not always possible due to patient re-allocation from hospitals and ICUs elsewhere in the Netherlands, and so for this study we included samples collected within 48 h after admission to the general ward, and 72 h after admission to the ICU. The delay in plasma collection may have influenced the results from this study.

To date, this is the largest study to investigate the relationship between adipokines and COVID-19. Here we show that plasma adipokine levels are associated with COVID-19 hospitalization, i.e., the need for oxygen support, or mechanical ventilation and other organ support in the ICU, but not mortality. Further studies are required to determine how the altered secretion of adipokines might influence severe inflammation, lung injury and respiratory failure in patients.

## Supplementary information

Supplementary Information

## Data Availability

The datasets generated during the current study are not yet publicly available but are available from the corresponding author on reasonable request.
