# Supplementary material for: Circulating adipokine levels and COVID-19 severity in hospitalized patients
Source: Int J Obes (Lond). 2022 Dec 12;47(2):126–37. doi: 10.1038/s41366-022-01246-5 (PMC9742670; doi:10.1038/s41366-022-01246-5)
Supplement: Supplementary file 1 — Supplementary Information [file 41366_2022_1246_MOESM1_ESM.pdf]

Supplementary Information for:

Circulating adipokine levels and COVID-19 severity in hospitalized patients
Flikweert et al.,

This pdf file includes:

Supplementary Figure 1: The number of symptom days COVID-19 patients had before admission to either the general ward or ICU during the 1st and 2nd wave of the COVID-19 pandemic.

Supplementary Figure 2: BMI distribution of included subjects separated by severity

Supplementary Table 1: Baseline patients' clinical characteristics, comorbidities, therapies and outcome stratified between low and high BMI based on the median BMI of hospitalized patients.(27.2kg/m^2).

Supplementary Figure 1.

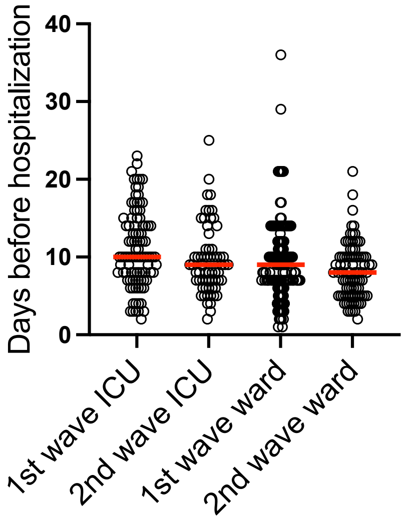

Supplemental figure 1. The number of symptom days COVID-19 patients had before admission to either the general ward or ICU during the 1st and 2nd wave of the COVID-19 pandemic. Each dot represents an individual patient. All patients in our cohort for which we had symptom information were included in this analysis (n=424), the red line indicates the median.

Supplementary Figure 2.

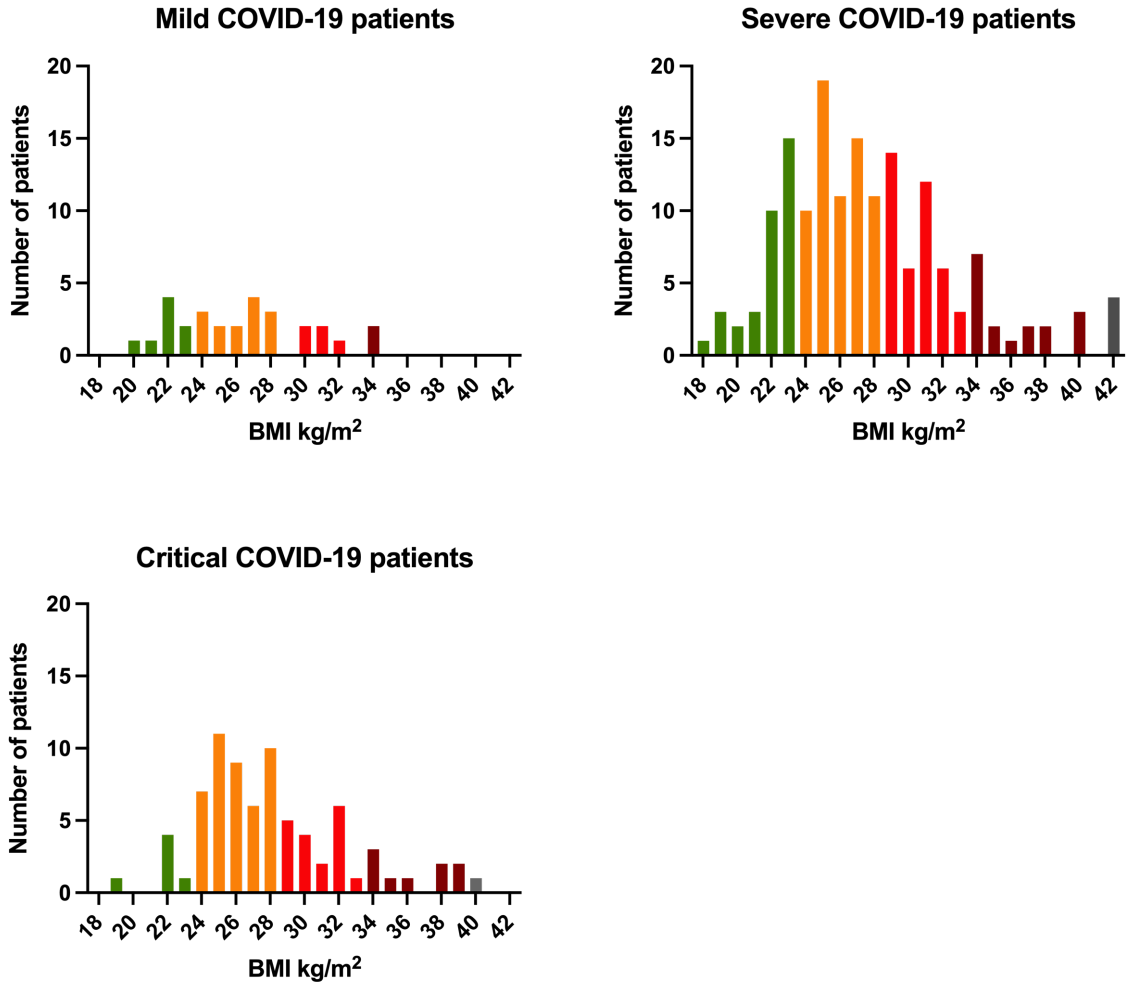

Supplemental Figure 2. BMI distribution of included subjects separated by severity; mild (home), severe (ward), and critical (ICU). The bar colours represent the extent of obesity based on the WHO classification. Green=normal weight, orange=overweight, red=obese and grey=morbid obese.

Supplemental Table 1. Baseline patients' clinical characteristics, comorbidities, therapies and outcome stratified between low and high BMI based on the median BMI of hospitalized patients.(27.2kg/m^2).

Variable	LOW BMI		HIGH BMI			
	**A** Severe COVID-19 **Low BMI** n= 83	**B** Critical COVID-19 **Low BMI** n= 32	**C** Severe COVID-19 **High BMI** n= 76	**D** Critical COVID-19 **High BMI** n= 39	**A vs B** *p* value	**C vs D** *p* value
**DEMOGRAPHICS**						
Age (years)	71 [58-79]	70 [63-75]	66 [54-71]	62 [56-72]	0.750	0.772
Male, n (%)	52 (62.7)	26 (81.3)	34 (44.7)	22 (56.4)	0.056	0.236
BMI (kg/m^2)	24.3 [22.7-25.6]	24.8 [23.7-25.9]	30.7 [28.7-33.6]	30.1 [28.3-33.6]	0.147	0.327
**COMORBIDITIES**						
Pulmonary disease, n (%)	13 (15.7)	9 (28.1)	22 (28.9)	9 (23.1)	0.128	0.502
Chronic cardiac disease, n (%)	25 (30.1)	10 (31.3)	15 (19.7)	11 (28.2)	0.906	0.304
Chronic kidney disease, n (%)	10 (12.0)	1 (3.1)	8 (10.5)	3 (7.7)	0.286	0.747
Hypertension, n (%)	28 (33.7)	10 (31.3)	27 (35.5)	15 (38.5)	0.800	0.757
Diabetes, n (%)	21 (25.3)	1 (3.1)	14 (18.4)	10 (25.6)	0.007	0.367
**CLINICAL COURSE**						
O2 need at hospital admission (L/min)	2 [0-4]	12 [3-15]	2 [0-4]	6 [3-15]	<0.001	<0.001
Hospital length of stay, days	5 [4-9]	16 [9-24]	5 [4-9]	18 [14-25]	<0.001	<0.001
**TREATMENT**						
Antiviral	13 (15.7)	6 (18.8	22 (28.9)	4 (10.3)	0.690	0.023
Antibiotics	54 (65.1)	32 (100)	45 (59.2)	39 (100)	<0.001	<0.001
Corticosteroids	26 (31.3)	16 (50)	28 (36.8)	15 (38.5)	0.062	0.865
Antifungal therapy	0 (0)	6 (18.8)	0 (0)	11 (28.2)	<0.001	<0.001
Chloroquine	41 (49.4)	17 (53.1)	32 (42.1)	22 (56.4)	0.720	0.146
**ICU CHARACTERISTICS**						
APACHE II score		18 [15-21]		15 [11-20]		
ICU length of stay, days		10 [5-21]		13 [8-25]		
Duration of MV, days		10 [4-20]		11 [7-24]		
PaO2/FiO2 after intubation		127 [101-151]		140 [96-201]		
Prone ventilation		19 (65.5)		28 (71.8)		
Tracheostomy		1 (3.1)		5 (12.8)		
ECLS		1 (3.1)		1 (2.6)		
**COMPLICATIONS**						
Pulmonary Embolism	4 (4.8)	11 (34.4)	1 (1.3)	7 (17.9)	<0.001	0.001
Pneumothorax	1 (1.2)	1 (3.1)	0 (0)	1 (2.6)	0.481	0.339
Stroke	1 (1.2)	5 (15.6)	0 (0)	2 (5.1)	0.006	0.113
Cardiac arrhythmia	4 (4.8)	11 (34.4)	2 (2.6)	5 (12.8)	<0.001	0.043
Cardiac ischemia	3 (3.6)	1 (3.1)	2 (2.6)	3 (7.7)	0.999	0.335
Bacteremia*	0 (0)	3 (9.4)	0 (0)	6 (15.4)	0.020	0.001
Acute kidney injury**	8 (9.6)	15 (46.9)	3 (3.9)	20 (51.3)	<0.001	<0.001
Renal replacement therapy	0 (0)	6 (18.8)	0 (0)	7 (17.9)	<0.001	<0.001
Liver dysfunction***	14 (16.9)	11 (34.4)	10 (13.2)	23 (59.0)	0.041	<0.001
**OUTCOME**						
Death		15 (46.9)		16 (41.0)		
**ADIPOKINES/CYTOKINES**						
Leptin, ng/mL	7.4 [3.1-14.3]	4.7 [1.9-9.5]	16.2 [7.6-33.5]	6.3 [4.0-24.5]	0.068	0.017
Adiponectin, μg/mL	3.9 [1.9-7.4]	2.8 [1.5-6.1]	3.4 [2.3-5.5]	2.6 [1.8-6.4]	0.114	0.337
Adiponectin/Leptin ratio	0.5 [0.2-1.6]	0.5 [0.3-1.5]	0.2 [0.1-0.4]	0.4 [0.2-0.7]	0.760	0.064
Resistin, ng/mL	18.1 [11.4-30.4]	24.0 [15.7-32.0]	18.5 [12.6-27.7]	21.5 [14.5-31.3]	0.139	0.229
Visfatin, ng/mL	3.7 [3.0-4.8]	4.7 [3.9-6.5]	3.8 [3.1-5.3]	4.5 [3.4-6.3]	0.002	0.113
IL-6, pg/mL	34.6 [19.6-49.9]	56.3 [35.2-113.6]	26.8 [21.1-42.0]	68.3 [36.5-124.1]	<0.001	<0.001
IL-10, pg/mL	10.1 [7.2-13.2]	11.7 [6.6-15.3]	9.8 [7.7-12.1]	12.3 [9.1-19.0]	0.389	0.005
TNF-α	50.9 [41.0-67.6]	70.3 [61.3-85.3]	60.5 [49.3-75.2]	72.9 [59.3-86.1]	<0.001	0.006
TNF-α /IL-10 ratio	5.8 [3.8-7.6]	6.4 [4.7-8.9]	5.9 [4.8-8.2]	5.2 [3.9-7.4]	0.176	0.155

Data are presented as median [IQR], or n and percentage. P-values are calculated using, Mann Whitney U, Chi-squared test or Fishers Exact Test. APACHE Acute Physiology And Chronic Health Evaluation, BMI body mass index, ECLS Extra Corporeal Life Support, ICU Intensive Care Unit. IL interleukin, MV mechanica ventilation, TNF tissue necrosis factor *positive blood cultures, **increase in serum creatinine by 26.5 umol/L within 48h, or >1,5 times baseline within the prior 7 days, ***an increase in blood bilirubin, alanine transaminase or aspartate transaminase that is twice the upper limit of the normal range.
